# How to Make Your Relationship Work? Aesthetic Relations with Technology

**DOI:** 10.1007/s10699-015-9449-4

**Published:** 2015-10-29

**Authors:** Jeannette Pols

**Affiliations:** 1 Section of Medical Ethics, Academic Medical Centre, Postbus 22700, 1100 DE Amsterdam, Netherlands; 2 Department of Anthropology, University of Amsterdam, Amsterdam, Netherlands

**Keywords:** Aesthetics, Technology, Affective relations, Ethics, Health care

## Abstract

Discussing the workings of technology in care as *aesthetic* rather than as ethical or epistemological interventions focusses on how technologies engage in and change relations between those involved. Such an aesthetic study opens up a repertoire to address values that are abundant in care, but are as yet hardly theorized. Kamphof studies the problem that sensor technology reveals things about the elderly patients without the patients being aware of this. I suggest improvement of these relations may be considered in aesthetic terms, for instance by developing the affective quality of people’s technological relationships.

Ike Kamphof’s beautiful paper proposes to understand the use of technology in care in terms of establishing aesthetic relations between nurses, patients and technologies. This is a clever way of taking the activity of technology seriously while avoiding technological determinism, at the same time creating space for the study and evaluation of relations that will have to be (re-)built in practice. The way these relations turn out to be is the result of the activities (and passivities) of *all* partners, and this can only be learned through careful empirical studies (Pols 2015). In Kamphof’s analysis the reader gets a convincing view on the aesthetic relational work of the *nurses*. The question I would like to explore further in this response is what the *technology* brings to the engagements.

Kamphof is not explicit about the ‘goods and beauties’ the sensor technology brings. She does not evaluate the stated goals of ‘staying at home longer’ by detecting possible problems through changes in movement patterns. She shows how the technology incidentally provided possibilities for patients to ‘save face’ when nurses could address problems they were hesitant to bring up. The question about the goals, goods and beauties of sensor technology is a crucial one for further study. Care for older people at home provides a context where solutions are anxiously anticipated, eagerly sold, and widely implemented. Little is known, however, about the possibilities for and shapes of the new relations they may help bring about.

So this is a first gain from Kamphof’s approach: it allows us to analyse ‘normativity in the making’. What also becomes clear is the problem that haunts the sensor project. Much of the work we see the nurses do is to *repair the* *flaws* the technology brings to the caring relations. The technology does not work as a device that assists the nurses to achieve the goals they had already formulated. It interferes with these goals by putting new possibilities, vistas and dilemmas on the care agenda. It is indeed a partner, and an unruly and unpredictable one at that. The core of these problems is that the carers know things about their patients, without the patients being aware of this. The patients’ movements are being registered and interpreted by the nurses, but the patients do not know this is happening. Kamphof lists 3 major problems with this and discusses a 4th one: (1) the patients may not want to discuss an event that the nurse has witnessed; (2) the patients may have a different interpretation of a situation than the nurse; (3) nurses may feel morally obliged ‘not to watch’ when they have seen things anyway; and (4) patients may feel disturbed when the nurse has been ‘watching’ them without them knowing this.

Why would we describe these problems as *aesthetic* problems? Can they be avoided or is a different aesthetic possible?

The meaning of aesthetics as a concept is not clear. Sometimes it is related to *epistemology*, to problems of ‘knowing’. At other times it is related to *ethics*, implying problems of *judging* and grounding judgment. There are also combinations between the two, as Lyotard makes clear in Kamphof’s paper. Evoking aesthetics by pointing to the relation to the senses often makes the connection to knowing, where visual metaphors have taken pride of place for knowing the world (Daston and Galison 2007). In the sensor case there are epistemological problems about what is—or should be—the object of knowing (Movements? Patients’ perceptions? Medical problems?).

The relation between aesthetics and ethics is often linked to justice or moral rightness rather than to ‘beauty’. Since Kant analytically assigned ethics and aesthetics to different domains, the latter have gradually become part of the sphere of the Fine Arts, and have disappeared from thoughts about daily life and care (Pols 2013). Meanwhile, aesthetic values are abundant in care (a beautiful scar, a wonderful nurse), as normative forms to express what is of value. They are, however, not often recognized and conceptualized in terms of aesthetics.

With her notion of ‘modest relational aesthetics’, Kamphof nicely shows the benefits of discussing care in aesthetic terms. She does this by linking it to ‘doing’, to the creation of new relations between people and technologies and the tinkering this involves. It is this situated, creative shaping of relations that makes this an aesthetic activity. It is modest in that it resembles the pragmatic work of a craftsperson, repairing a chair rather than creating a painting. It is normative, but oriented towards situated aesthetic values that may differ between people and situations, rather than to principles that count for everyone, everywhere. A ‘fit’ needs to be actively achieved.

When looking at aesthetics in this way, the problem of ‘seeing the patient without the patient being aware’ can be seen as an aesthetic problem: it hinders the creation of relations that are aesthetically pleasing or good, and may threaten good relations that were already negotiated and established. This of course relates to epistemological issues (what can be known by monitoring movements?) and ethical norms and principles such as ‘one should not lie and deceive’ (Schermer 2007). Yet it is attractive to use the lens of relational aesthetics to study locally how relations turn out in terms of trust, harmony, and helping patients to achieve a good life rather than disturbing it. This is a practical and relational accomplishment oriented towards aesthetic values.

The question is then if aesthetic improvement is possible in the case of the sensors. An improvement strategy could be to take more seriously the notion that people and technology engage in relations with one another. Often, these affective relations make the use of certain devices successful, but for different reasons then originally intended (Pols and Moser 2009). People come to love their telecare devices or robot pets, and the ways the devices organize relations with the nurse or with fellow patients. In the sensor case this ability is lost or greatly reduced for the patients. They cannot relate well to the sensors, because the sensors have been designed to be almost invisible. Apart from creating the problems discussed, this is also a waste of the potential of technology to take part in caring relations. When people can *see* they are being watched by a visible and attractive technology, they may feel safer and more connected to their nurses. From focus groups on telecare technologies we conducted with older people, the participants unanimously feared being left alone, and also unanimously discounted privacy in favor of ‘being watched’ and hence ‘looked after’. The vision of lying helplessly on the floor with nobody to rescue them changed their idea of privacy as a value into privacy as a potential nightmare.

If people engage in affective relations with and through technologies—and the passionate relations with and through mobile phones, tablets and computers are clear examples of this—making the monitoring more overt seems to present a moral and aesthetic win–win situation: no explicit and intended misleading of the patient, and strengthening rather than jeopardizing meaningful relations. ‘Scottie’, developed by the Waag Society, is an example. Scottie was designed to create virtual closeness between hospitalized children and their parents. The device shows light in different colors to communicate moods of the sender.
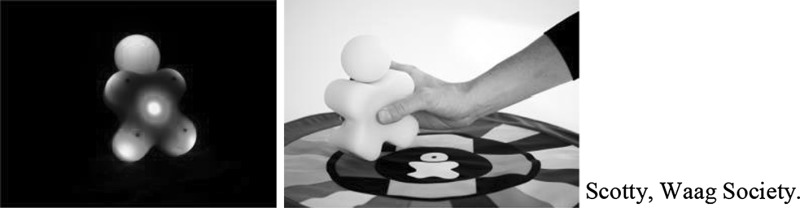
Although people with cognitive problems may not always realize what that nice warm light represents, they may come to connect it to their carers and feel safe by its presence. *Whether* this will work out like this, remains to be tested, but in one of Kamphof’s other papers she describes how one of the patients symbolically waved to her not-so-visible sensors before she went to bed. It was her way to say good-night to the nurse and acknowledge their connection through the technology.

Making the sensors into tangible nice things to relate to will not completely solve problems of ‘the nurse knowing more than you do’. People will forget they are being monitored, and there will always be translation problems in understanding ‘movement patterns’ in terms of ‘care needs’. Yet it would provide patients with possibilities to relate to the technology more actively and create more aesthetically pleasing relations between participants and technology.

It is high time for experiments in care, and careful ethnographic descriptions of the type of aesthetics these may lead to.
